# Beyond the Well-Known Phytochemicals: Cryptic Plant-Derived Histone Acetyltransferase (HAT) and Histone Deacetylase (HDAC) Modulators as Epigenetic Adjuvants in Cancer Therapy

**DOI:** 10.3390/cells15212035

**Published:** 2026-11-08

**Authors:** Estera Okoń, Agata Jarząb, Wirginia Kukula-Koch, Joanna Tokarczyk, Wojciech Koch, Anna Boguszewska-Czubara, Anna Wawruszak

**Affiliations:** 1 Department of Biochemistry and Molecular Biology, Medical University of Lublin, 1 Chodzki Str., 20-093 Lublin, Poland; estera.okon@umlub.edu.pl (E.O.); agata.jarzab@umlub.edu.pl (A.J.); 2 Department of Pharmacognosy with Medicinal Plants Garden, Medical University of Lublin, 1 Chodzki Str., 20-093 Lublin, Poland; virginia.kukula@gmail.com; 3 Department of Food and Nutrition, Medical University of Lublin, 4a Chodźki Str., 20-093 Lublin, Poland; tokarczyk.joanna@outlook.com (J.T.); kochw@interia.pl (W.K.); 4 Department of Medical Chemistry, Medical University of Lublin, 4a Chodźki Str., 20-093 Lublin, Poland; anna.boguszewska-czubara@umlub.edu.pl

**Keywords:** histone acetyltransferases (HATs), histone deacetylases (HDACs), parthenolide, anacardic acid, formononetin, plumbagin, allicin, plant-derived adjuvants, cancer therapy

## Abstract

**Highlights:**

**What are the main findings?**

- Plant-derived phytochemicals, including parthenolide, anacardic acid, formononetin, plumbagin, and allicin/S-allylmercaptocysteine (SAMC), can modulate histone acetyltransferase (HAT) and histone deacetylase (HDAC) expression/activity through distinct molecular mechanisms.
- These natural compounds may affect chromatin accessibility, gene expression, and cancer-related signaling, highlighting underexplored plant-derived HAT/HDAC modulators as potential epigenetic adjuvants.

**What are the implications of the main findings?**

- Targeting HAT/HDAC-dependent epigenetic mechanisms with phytochemicals may provide complementary strategies for enhancing the efficacy of conventional and targeted anticancer therapies.
- Further mechanistic, pharmacological, and translational studies are needed to establish their selectivity, bioavailability, safety, and potential for clinical application as epigenetic adjuvants.

**Abstract:**

Epigenetic dysregulation constitutes a fundamental molecular hallmark of oncogenesis, arising from aberrant alterations in chromatin architecture and transcriptional regulation that disrupt cellular differentiation, sustain malignant phenotypes, promote therapeutic resistance, and facilitate tumor initiation and progression. Among the principal epigenetic mechanisms, histone acetylation is dynamically regulated by histone acetyltransferases (HATs) and histone deacetylases (HDACs), making these enzymes attractive targets for pharmacological intervention. Although several synthetic epigenetic drugs have entered clinical practice, their therapeutic utility may be constrained by limited selectivity, toxicity, and adaptive resistance, prompting the continued investigation of structurally diverse natural compounds. The review examines comparatively underexplored plant-derived phytochemicals with reported or proposed HAT- and/or HDAC-modulatory properties, focusing on parthenolide, anacardic acid, formononetin, plumbagin, and allicin and its derivatives, including S-allylmercaptocysteine (SAMC). The available evidence indicates that these compounds can influence epigenetic processes through distinct, pleiotropic, mechanisms, potentially affecting cellular proliferation, apoptosis, differentiation, and responses to anticancer treatment. Collectively, these findings support the further investigation of cryptic plant-derived HAT/HDAC modulators as candidate epigenetic adjuvants, while underscoring the need for rigorous mechanistic validation, pharmacokinetic characterization, and translational assessment.

## 1. Introduction

**Figure 1:**
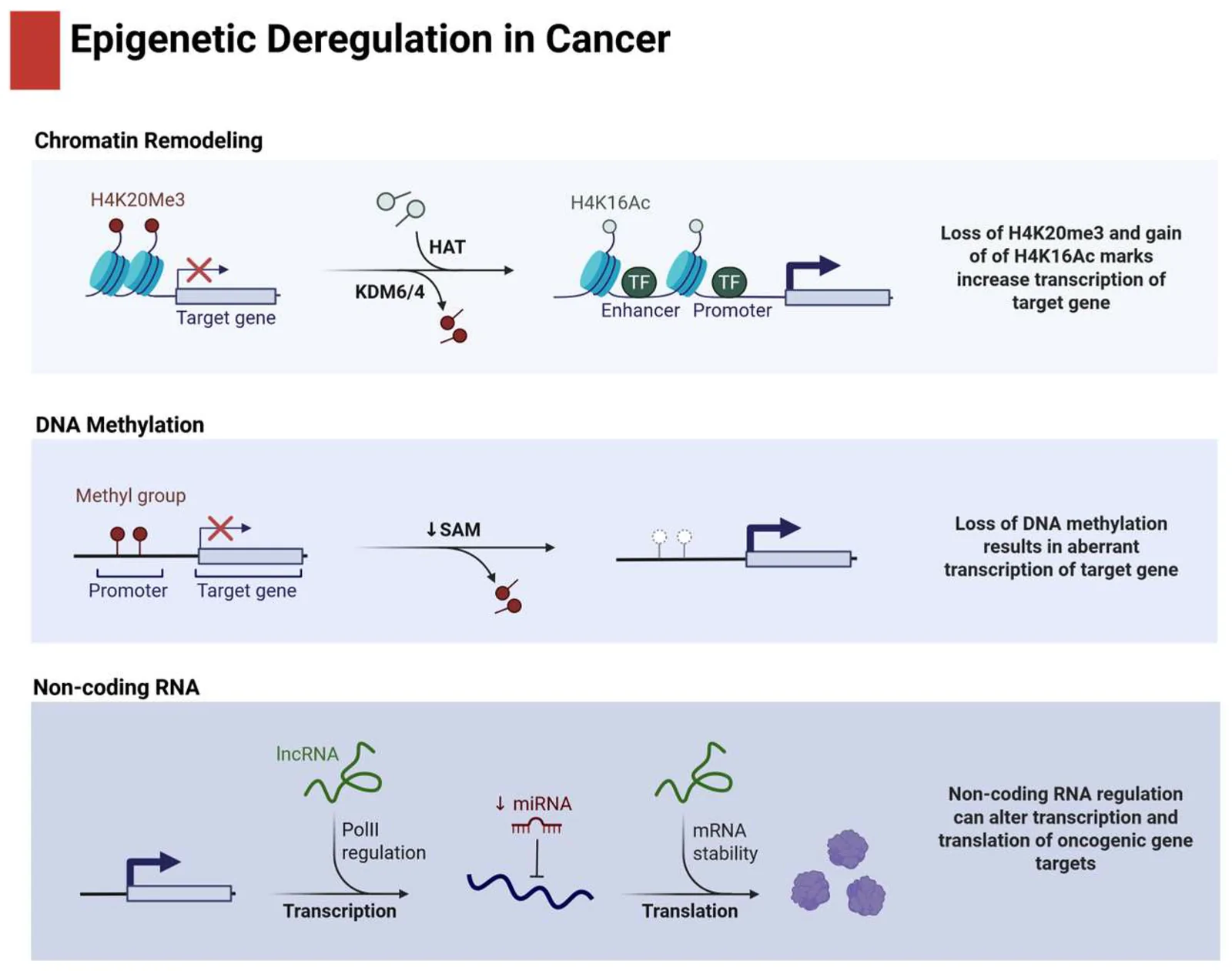
Epigenetic deregulation in cancer. Schematic overview of the major epigenetic mechanisms involved in cancer development and progression, including chromatin remodeling, DNA methylation, and non-coding DNA/RNA regulation. The figure was created in Biorender. Estera Okoń et al. (2026) https://BioRender.com.

Natural products derived from diverse biological sources, including higher plants [1], microorganisms [2], and marine sponges [3], constitute a relevant reservoir of bioactive compounds capable of modulating epigenetic processes [4]. Epigenetics encompasses stable, heritable alterations in gene expression and chromatin organization that occur independently of changes in the underlying DNA nucleotide sequence [4]. These processes are mediated by a complex network of epigenetic regulators, including DNA methyltransferases [5], histone acetyltransferases [6] and deacetylases [7], histone lysine [8] and arginine methyltransferases [9], as well as histone demethylases [10]. The principal epigenetic regulatory mechanisms comprise DNA methylation [11], chromatin remodeling [12], post-translational modifications of histones [13], and non-coding RNA-mediated transcriptional and post-transcriptional gene silencing (Figure 1) [14].

Dysregulation of these mechanisms may result in aberrant patterns of gene expression and chromatin organization, thereby contributing to the molecular pathogenesis of a broad spectrum of diseases, including cancer [15], neurodegenerative disorders [16], and metabolic diseases [4].

Plant-derived secondary metabolites constitute a chemically and structurally diverse, yet still insufficiently characterized, reservoir of epigenetic modulators with considerable potential for cancer therapy [17]. Within this emerging group of rare and comparatively underexplored phytochemicals, parthenolide [18], anacardic acid [19], formononetin [20], plumbagin [21], allicin [22], and S-allylmercaptocysteine (SAMC) [23] have attracted increasing attention due to their reported capacity to modulate HDAC activity, histone acetylation, and downstream transcriptional networks implicated in malignant transformation and tumor progression. Despite their distinct chemical scaffolds and botanical origins, the epigenetic mechanisms underlying their biological activity remain incompletely elucidated and may involve both direct interference with HDAC function and the indirect regulation of HDAC-dependent signaling pathways. Accordingly, the present review examines these poorly characterized plant-derived compounds, with particular emphasis on their chemical classification, natural occurrence, molecular mechanisms of HAT/HDAC modulation, and their prospective application as novel epigenetic adjuvants in cancer therapy [17].

## 2. Histone Acetyltransferases (HATs) and Histone Deacetylases (HDACs)

**Figure 2:**
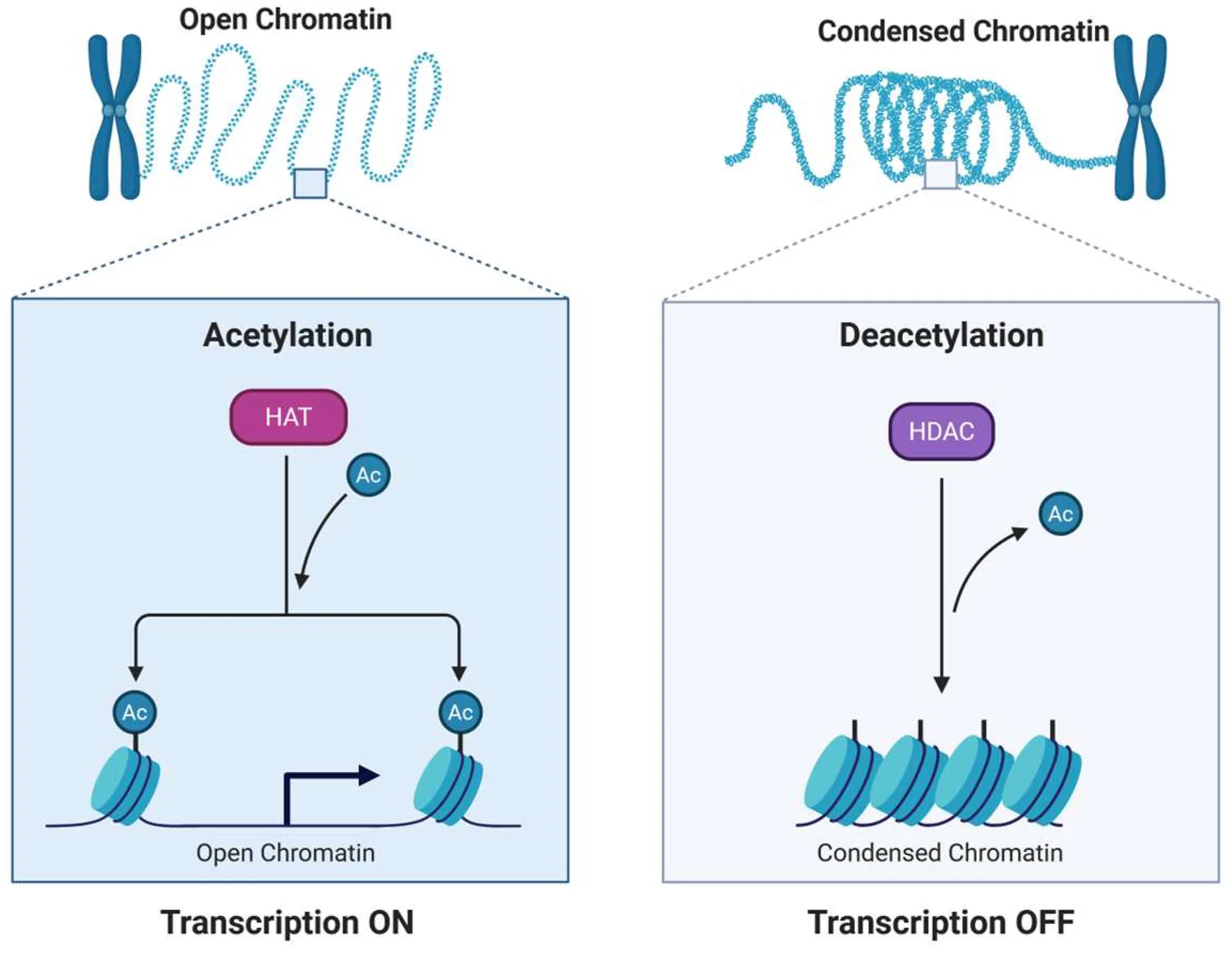
Histone acetyltransferase (HAT) and histone deacetylase (HDAC) activity. Schematic representation of HAT and HDAC activities and their roles in the regulation of chromatin structure and gene expression. The figure was created in Biorender. Estera Okoń et al. (2026) https://BioRender.com.

The dynamic equilibrium between protein acetylation and deacetylation constitutes a fundamental regulatory mechanism governing chromatin organization, gene expression, and diverse cellular processes. This balance is maintained predominantly through the coordinated activities of histone acetyltransferases (HATs) [24] and histone deacetylases (HDACs) [25], which function within interconnected regulatory networks rather than as strictly independent opposing enzymes (Figure 2) [26].

The functional implications of histone acetylation extend beyond the canonical framework that primarily associates acetylation with transcriptional activation and deacetylation with transcriptional repression [27]. Accumulating evidence indicates that the functional consequences of histone acetylation are highly context-dependent and are determined by the specific chromatin environment, the identity and genomic localization of the modified histone residue, as well as the coordinated activity of the molecular machinery responsible for the recognition, deposition, and removal of these epigenetic changes [28]. Perturbation of the HAT/HDAC balance has been implicated in tumor initiation and progression, contributing to aberrant transcriptional programs characteristic of malignant cells [29]. The pharmacological inhibition of HDACs promotes the accumulation of acetylated lysine residues on core histones and can facilitate the reactivation of transcriptionally silenced regulatory genes, providing a mechanistic basis for the antitumor activity of HDAC inhibitors (HDIs) [30]. In this context, increasing evidence indicates that naturally occurring bioactive compounds derived from vegetables, fruits, whole grains, and microorganisms can modulate HDAC activity and may contribute to chemopreventive and therapeutic responses [31]. These findings have consequently stimulated interest in both dietary and non-dietary natural HDAC modulators, alongside synthetic HDIs, as potential components of anticancer strategies, including their use in combination with conventional chemotherapy or radiotherapy [26].

### 2.1. Histone Acetyltransferase (HAT) Classification

Histone acetyltransferases (HATs), also referred to as lysine acetyltransferases (KATs), comprise a functionally and structurally heterogeneous group of enzymes responsible for the transfer of acetyl groups to lysine residues of histone and non-histone proteins [32]. Based on their subcellular localization and substrate specificity, HATs have traditionally been divided into type A and type B acetyltransferases [33]. Type B HATs are predominantly cytoplasmic and catalyze the acetylation of newly synthesized, free histones prior to their nuclear translocation and incorporation into nascent chromatin. In contrast, type A HATs are primarily nuclear and function within multiprotein regulatory complexes that target nucleosomal histones [34]. Their catalytic activity modulates chromatin accessibility by altering histone–DNA interactions and thereby facilitates transcriptional regulation and DNA damage responses. Type A HATs can be further classified according to sequence homology, domain organization, and catalytic architecture into several major families. These include the GNAT (Gcn5-related N-acetyltransferase) family, represented by GCN5/KAT2A and PCAF/KAT2B; the MYST family, comprising, among others, TIP60/KAT5 and MOZ/KAT6A; the p300/CBP family, encompassing EP300/KAT3B and CREBBP/KAT3A; and the SRC/NCoA (KAT13) family of nuclear receptor coactivators [35]. Collectively, these enzymes act as key epigenetic “writers” by establishing acetylation marks that regulate chromatin architecture, transcriptional activity, DNA repair, and cellular identity [36]. Importantly, their biological functions are determined not only by catalytic activity but also by the modular domain architecture and protein–protein interactions that enable their recruitment to specific genomic loci and integration into context-dependent regulatory complexes [32].

### 2.2. Histone Deacetylase (HDAC) Classification

Histone deacetylases (HDACs) constitute a family of epigenetic enzymes that catalyze the removal of acetyl groups from lysine residues within histone tails and a broad spectrum of non-histone substrates. Through modulation of lysine acetylation, HDACs contribute to the dynamic regulation of chromatin architecture and transcriptional activity [37]. In the canonical model, HDAC-mediated deacetylation promotes chromatin condensation by increasing histone–DNA interactions, thereby restricting the accessibility of transcriptional regulatory factors and contributing to transcriptional repression. However, the biological functions of HDACs extend well beyond their canonical role in chromatin remodeling, encompassing the regulation of diverse cellular processes, including cell-cycle progression [38], apoptotic pathways [39], inflammatory and immune signaling [40], redox homeostasis [41], as well as neuronal differentiation and function [42,43]. The dysregulation of HDAC activity has been associated with pathological alterations in gene expression and cellular signaling, particularly in cancer and neurodegenerative disorders, establishing these enzymes as pharmacologically relevant epigenetic targets. Consequently, HDIs have emerged as important epigenetic modulators capable of altering aberrant acetylation states and reshaping transcriptional programs. Pharmacological inhibition of HDACs may restore the expression of transcriptionally silenced genes and interfere with signaling networks that sustain pathological cellular phenotypes [37]. Depending on their molecular selectivity and cellular context, HDIs have demonstrated antiproliferative [44], pro-apoptotic [45], anti-inflammatory [46], immunomodulatory [46], and neuroprotective activities [47] in experimental models. Accordingly, accumulating preclinical data and emerging clinical evidence support the continued investigation of HDAC-targeted strategies across a spectrum of malignancies and neurological disorders, while also emphasizing the importance of isoform selectivity, tissue specificity, and context-dependent biological responses in the development of next-generation HDAC-directed therapeutics [37].

**Figure 3:**
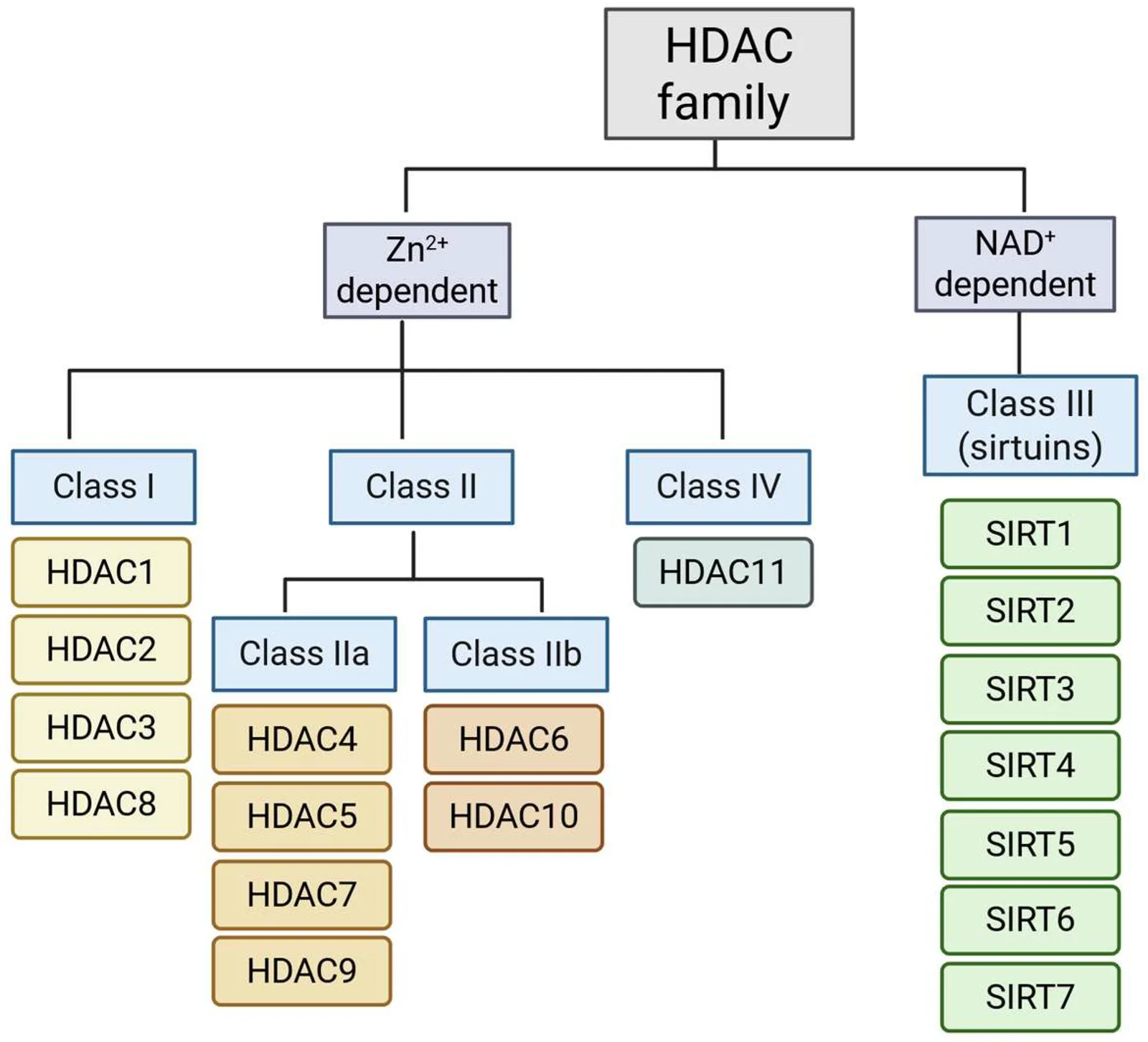
Histone deacetylase (HDAC) classification. Classification of HDACs into four major classes based on their structural characteristics, cellular localization, and sequence homology. The figure was created in Biorender. Estera Okoń et al. (2026) https://BioRender.com.

Mammalian HDACs comprise a structurally and functionally heterogeneous enzyme superfamily that can be broadly classified according to sequence homology, catalytic domain architecture, and cofactor requirements (Figure 3).

The canonical zinc-dependent HDACs are organized into class I, class II, and class IV, whereas the sirtuins constitute class III HDACs, which are mechanistically distinct and require nicotinamide adenine dinucleotide (NAD^+^) as a cosubstrate for catalytic activity [48]. Class I HDACs, comprising HDAC1, HDAC2, HDAC3, and HDAC8, exhibit the highest sequence similarity to the corresponding yeast deacetylases and are predominantly nuclear [49]. Class II HDACs are further subdivided into class IIa (HDAC4, HDAC5, HDAC7, and HDAC9) and class IIb (HDAC6 and HDAC10) based on their domain organization, subcellular distribution, and substrate specificity [50]. HDAC11, the sole member of class IV, represents a distinct zinc-dependent deacetylase group with unique structural and functional characteristics [51]. In contrast, class III HDACs, comprising SIRT1-SIRT7, constitute the evolutionarily conserved sirtuin family and are distinguished by their dependence on NAD^+^ as a cosubstrate, catalyzing deacylation reactions that functionally couple post-translational protein modification to the cellular metabolic state [52].

Collectively, the mammalian HDAC superfamily comprises 18 enzymes segregated into four principal classes, exhibiting extensive molecular, structural, and functional heterogeneity with respect to subcellular compartmentalization, substrate selectivity, regulatory architecture, catalytic properties, and physiological functions. This pronounced heterogeneity constitutes a critical determinant of the context-specific biological consequences arising from aberrant HDAC activity and represents a fundamental consideration in the rational development of selective, isoform- and context-directed HDAC-targeted therapeutic interventions [48].

### 2.3. Ongoing Clinical Development of HDAC/HAT Therapeutics

The clinical development of epigenetic therapeutics targeting histone acetylation is substantially more advanced for HDAC than for HAT inhibitors. Several HDACs, including vorinostat, panobinostat, belinostat and romidepsin, have received regulatory approval for selected hematological malignancies. Nevertheless, their clinical utility remains constrained by treatment-related toxicity and limited efficacy in solid tumors [53,54]. In contrast, the clinical development of HAT inhibitors has remained comparatively limited due to low selectivity and the lack of pharmacological specificity. A notable advancement in this field is OP-3136, an orally bioavailable inhibitor targeting lysine acetyltransferases KAT6A and KAT6B, which has entered first-in-human phase I clinical evaluation in patients with advanced or metastatic solid tumors. This study is designed to characterize its safety profile, tolerability, pharmacokinetic and pharmacodynamic properties, and preliminary antitumor activity, including its potential application in combination with endocrine therapies [55]. Importantly, the therapeutic targeting of KAT6A/KAT6B should be mechanistically distinguished from the inhibition of other acetyltransferases, particularly p300/CBP, given their distinct substrate specificities, regulatory functions, and implications for tumor biology. Collectively, these developments underscore the need for the rational design of isoform-selective inhibitors, the identification of reliable pharmacodynamic biomarkers, and the systematic validation of target engagement and antitumor efficacy to facilitate the clinical translation of HAT-directed therapeutics.

## 3. Plant-Derived HAT and HDAC Modulators

Bioactive compounds obtained from plant sources have been used in medicine for decades owing to their diverse biological activities and therapeutic properties. Accumulating evidence indicates that natural products constitute a valuable and still insufficiently explored reservoir of pharmacologically active compounds. Their remarkable structural diversity and ability to modulate key molecular pathways involved in carcinogenesis make them promising candidates for the discovery and development of novel anticancer agents, as well as compounds with potential applications in cancer chemoprevention [56]. A particularly promising area of research concerns plant-derived modulators of HATs and HDACs, which may influence the epigenetic regulation of gene expression and thereby affect key cellular processes involved in tumor initiation and progression [57].

### 3.1. Parthenolide (PTL)

**Figure 4:**
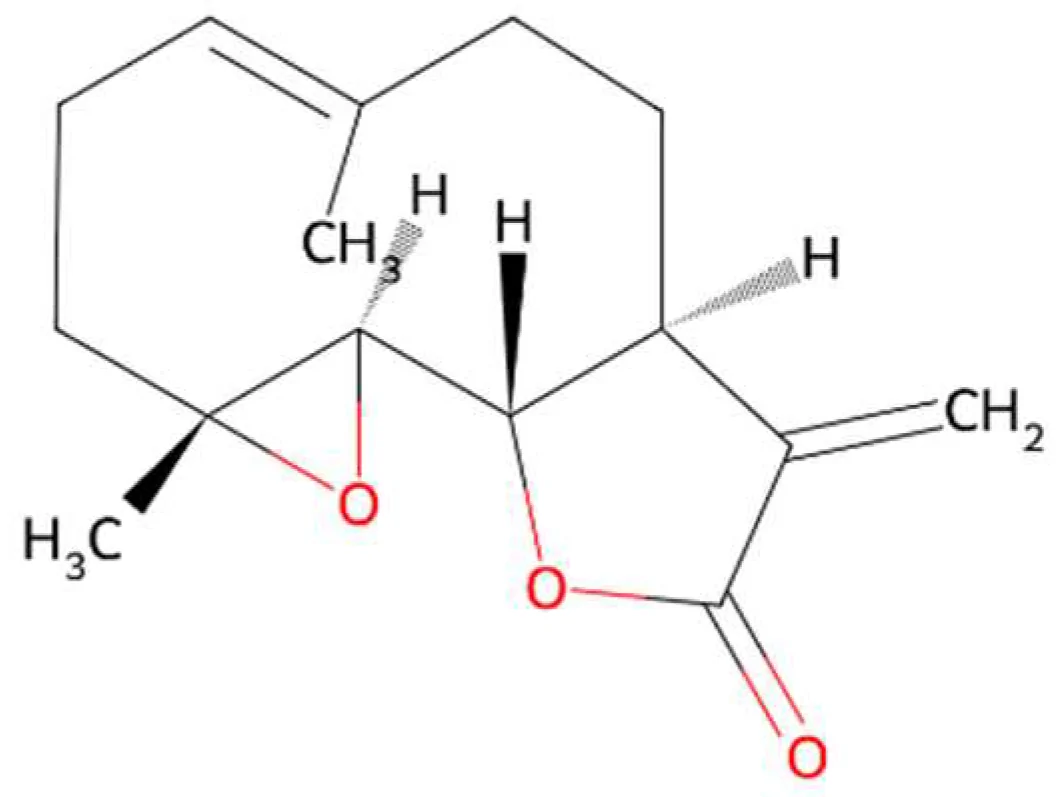
The chemical structure of parthenolide.

Parthenolide (PTL) is a plant-derived germacranolide sesquiterpene lactone characterized by an α-methylene-γ-lactone moiety and an epoxide within its ten-membered carbocyclic scaffold (Figure 4) [58].

Its best-characterized botanical source is feverfew, *Tanacetum parthenium* (L.) Sch.Bip. (Asteraceae), in which it occurs predominantly in the aerial parts, particularly the leaves and flower heads, whereas concentrations in the roots are low or undetectable [59]. PTL has also been reported from other Tanacetum species, including *T. larvatum* and *T. vulgare*, and was isolated from *Magnolia grandiflora* (Magnoliaceae) in an early phytochemical investigation [60,61,62].

The ethnomedicinal history associated with PTL pertains primarily to feverfew preparations rather than the isolated compound. Feverfew has traditionally been used for fever and headache, including migraine, as well as rheumatic pain, gastrointestinal discomfort, menstrual complaints and toothache; historical accounts additionally describe its use during childbirth and as a remedy for insect bites [58,59]. These uses establish the botanical context in which PTL was identified but should be distinguished from evidence that purified PTL is clinically effective for any of those indications.

A range of scientific publications prove that PTL exhibits anticancer activity through several complementary mechanisms, including the suppression of prosurvival signaling, induction of oxidative stress and mitochondrial apoptosis, and disruption of mitosis. In primary human acute myeloid leukemia (AML), exposure to 5–7.5 μM PTL for 18 h preferentially eliminated malignant cells, including primitive CD34^+^CD38^−^ populations, while largely sparing normal haematopoietic cells under the same experimental conditions. These effects were associated with the inhibition of NF-κB, increased reactive oxygen species generation, and the activation of p53; moreover, ex vivo treatment with 7.5 μM PTL reduced subsequent AML engraftment in NOD/SCID mice, supporting activity against functional leukemia stem cells [63]. Anticancer activity has also been demonstrated in solid tumor models. In HT-29, SW620, and LS174T colorectal cancer cells, 20 μM PTL for 24 h reduced the MTT-based viability signal by approximately 50%. Mechanistic experiments in HT-29 cells demonstrated mitochondrial depolarisation, reduced Bcl-2 expression, increased truncated Bid, mitochondrial redistribution of Bax, cytochrome c release, and caspase-3 activation. In the corresponding HT-29 xenograft model, intraperitoneal administration of 4 mg/kg three times weekly, beginning five days after tumor implantation, suppressed tumor growth and reduced tumor vascularisation while increasing apoptosis [64]. More recently, PTL was shown to covalently modify Cys54 of the kinetochore protein ZNF207/BUGZ through its α-methylene-γ-lactone moiety, impairing kinetochore–microtubule attachment and chromosome alignment and thereby identifying an additional mechanism underlying its antimitotic activity [65].

Particularly relevant to epigenetic combination therapy, PTL enhanced the cytotoxicity of the HDAC inhibitors vorinostat and panobinostat in AML models, including primary blasts. The 24 h experiments performed with 4 μM PTL with 1.5 μM vorinostat or 10 nM panobinostat in primary AML cells showed a potentiation involved suppression of treatment-induced NF-κB activation and MKK7-dependent activation of JNK1 [66]. For the moment, the translation of these findings is constrained by systemic exposure. In a phase I study in patients with cancer, an oral feverfew preparation providing 1–4 mg PTL daily in 28 day cycles was well-tolerated, but plasma PTL remained below the assay detection limit of 0.5 ng/mL (Table 1) [67]. Thus, the available findings support further investigation of PTL as an anticancer and chemosensitising candidate, while the experimental concentrations and animal doses should not be interpreted as established therapeutic regimens in humans.

PTL is best considered a pleiotropic, plant-derived HDAC modulator and a candidate epigenetic combination partner. The association between PTL and HDAC regulation was established by Gopal et al. [68], who reported selective depletion of HDAC1 protein without comparable effects on the other class I/II HDACs examined. HDAC1 loss involved proteasomal degradation and depended on ataxia telangiectasia mutated (ATM), rather than IKK2, a previously recognized PTL target. The resulting transcriptional changes resembled those elicited by pan-HDAC inhibitors [68]. These findings support modulation of HDAC1 abundance rather than, by themselves, direct inhibition of its catalytic site.

In another study, a significant inhibition of HDAC activity by PTL at the concentrations of 5 and 20 µM was proved. The experiment was performed in a cell-free fluorometric assay using a crude HDAC preparation, whereas vorinostat and trichostatin served as positive controls. Also, molecular docking against HDAC2 provided complementary computational evidence for the activity of this sesquiterpene lactone [69]. It is worth noting that this study did not establish an experimentally measured HDAC1-specific IC_50_ or an isoform-selectivity profile.

The epigenetic effects of PTL are also context-dependent. In the JB6P+ epidermal tumor-promotion model, Ghantous et al. [70] observed an increased p21 expression and altered chromatin accessibility at its promoter without changes in global histone acetylation, histone methylation, or overall HDAC activity [70]. More recently, Tang et al. [71] showed that 4 µM PTL for three days reduced HDAC1 protein levels in U87 and A172 glioma cells, alongside the appearance of neuronal markers and reduced proliferation. Nevertheless, the contribution of HDAC1 downregulation to these phenotypic changes remained a proposed mechanism [71].

The available scientific evidence indicates that the compound may also exhibit considerable therapeutic potential when administered in combination with established HDAC inhibitors, suggesting possible synergistic or additive effects that could enhance the overall efficacy of epigenetic anticancer strategies. In human acute myeloid leukemia models, PTL enhanced apoptosis induced by vorinostat and panobinostat, including primary AML blasts. Mechanistic experiments implicated the suppression of treatment-induced NF-κB signaling and MKK7-dependent JNK1 activation [66]. In MDA-MB-231 triple-negative breast cancer cells, Carlisi et al. [72] reported synergy following SAHA pretreatment and subsequent combined exposure to SAHA and PTL. The combination opposed protective signaling responses and retained SAHA-associated histone H3/H4 hyperacetylation and PTL-associated DNMT1 downregulation [72]. Thus, the histone changes in this combination cannot be assigned to PTL alone.

Other combinations extend this preclinical rationale. In Daoy and D283 Med medulloblastoma cells, PTL combined with 5-aza-2′-deoxycytidine reduced viability synergistically, although the range of synergistic responses differed between the cell lines [73]. In murine ASZ basal cell carcinoma cells, PTL reduced Gli1 expression and cooperated with an atypical protein kinase C inhibitor to suppress cell growth [74]. These observations support combination testing but do not establish that all antitumour effects are mediated by HDAC1. The sesquiterpene lactone also affects DNA methylation and DNMT1 regulation, providing a separate epigenetic mechanism [75].

Additionally, the water-soluble analog—dimethylaminoparthenolide (DMAPT)—has shown additional epigenetic effects, including cell type-dependent HDAC1 downregulation [76], opening the doors to studies on the derivatives of this molecule. In the case of PTL, certainly, the available evidence supports further mechanistic and pharmacological investigation, rather than established clinical efficacy as an HDAC-directed cancer adjuvant. Nevertheless, these initial findings shed light on its actual potential.

### 3.2. Organosulfur Compounds (alliin, allicin, S-allylcysteine (SAC), S-allylmercaptocysteine (SAMC), Diallyl Sulfide (DAS), Diallyl Disulfide (DADS), Diallyl Trisulfide (DATS))

**Figure 5:**
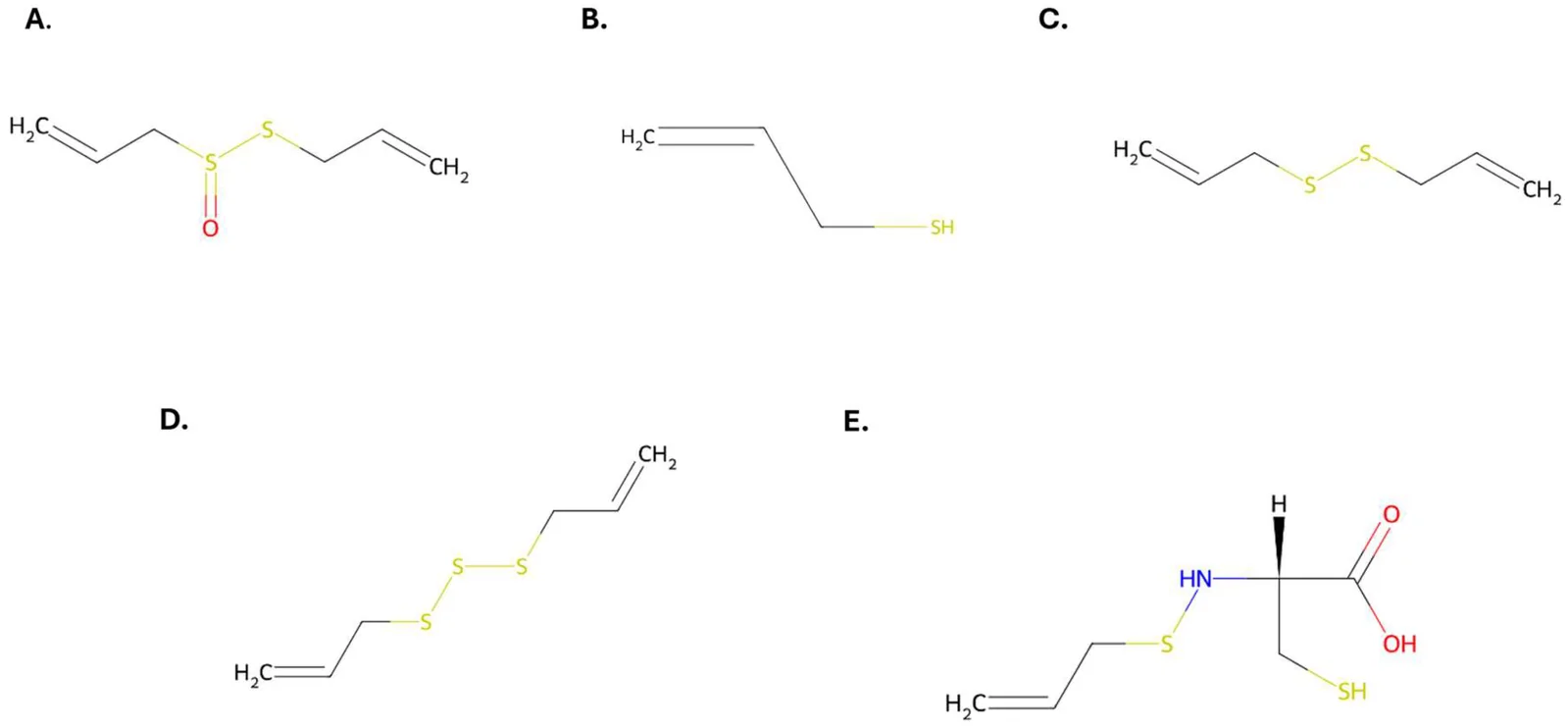
The chemical structure of organosulfur compounds ((**A**). Allicin; (**B**). Allyl mercaptan (AM); (**C**). Diallyl disulfide (DADS); (**D**). Diallyl trisulfide (DATS); (**E**). S-allylomercaptocysteine (SAMC)).

Known for centuries not only for its culinary applications but also for its therapeutic properties, garlic contains several biologically active organosulfur compounds (Figure 5) [77]. Allicin is the major lipid-soluble, unstable diallylthiosulfinate formed from the non-proteinogenic amino acid alliin by the action of alliinase in damaged tissues of plants belonging to the Amaryllidaceae family, including garlic *(Allium sativum)* and wild garlic *(Allium ursinum)* [78]. The biological activity of allicin is attributed to the high reactivity of its thiosulfinate group, which enables the oxidation of thiol groups in cysteine and glutathione. This process leads to alterations in protein structure, including the formation of disulfide bonds, resulting in the loss or acquisition of specific protein functions [79,80,81].

The anticancer effects of allicin are well-documented and are primarily associated with the induction of apoptosis. This process involves the disruption of mitochondrial membrane integrity, activation of both caspase-dependent and caspase-independent pathways, upregulation of pro-apoptotic proteins (Bax and Bad), downregulation of anti-apoptotic proteins (Bcl-2 and Bcl-xL), activation of the tumor suppressor protein p53, and modulation of signaling pathways involved in cancer cell survival [77,82,83]. Since the inhibition of HDACs represents one of the mechanisms capable of triggering apoptosis [9,10], these findings may suggest a potential role for allicin in the regulation of HDAC activity. However, the direct inhibition of HDACs by allicin has not yet been demonstrated. Lea et al. [84] reported increased levels of mono-, di-, and tri-acetylated histones in DS19 murine erythroleukemia cells following 24 h of allicin treatment, although it was not established whether this effect resulted from direct HDAC inhibition [84]. Moreover, allicin is highly unstable, exhibiting a half-life of less than 1 min under physiological conditions [85]. It is rapidly converted into allyl mercaptan (AM) as well as 2-ethenyl-4H-1,3-dithiin and diallyl disulfide DADS [86]. In the presence of cysteine, S-allylmercaptocysteine (SAMC) is also formed [87]. In addition, the highly reactive allylthio group promotes rapid autoxidative degradation, providing the rationale for developing strategies to improve allicin stability, including the use of nanocarrier-based delivery systems [77,88,89]. In addition to alliin and allicin, garlic contains numerous other organosulfur compounds, including S-allylcysteine (SAC), S-allylmercaptocysteine (SAMC), diallyl sulfide (DAS), diallyl disulfide (DADS), and diallyl trisulfide (DATS). These compounds are metabolized into allyl mercaptan (AM), allyl methyl sulfide (AMS), and methyl mercaptan [90]. Some of these metabolites are discussed below in the context of their potential to inhibit HDAC activity.

Allyl mercaptan (AM) is the most extensively characterized garlic-derived metabolite exhibiting HDAC inhibitory activity [90]. In DS19 murine erythroleukemia cells, AM increased histone acetylation and, at a concentration of 0.5 mM, reduced HDAC activity by 74% [91]. The authors suggested that the allyl group plays a crucial role in this effect. This hypothesis was further supported by molecular docking studies demonstrating that the small AM molecule readily accommodates within the active site of HDAC8, where it forms a strong sulfur–zinc interaction as well as favorable hydrophobic interactions [92]. In the studies conducted by Nian et al. [93], among the compounds tested (DADS, SAC, SAMC, allyl methyl sulfide, and AM), only AM demonstrated clear, dose-dependent HDAC inhibition in a cell-free system, with an IC_50_ value of 20 µM. In HT-29 colon cancer cells, AM treatment resulted in hyperacetylation of histone H3 at the P21WAF1 gene promoter and increased binding of the transcription factor Sp3 after only 4 h of exposure. After 24 h, enhanced recruitment of the p53 protein to the regulatory region of this gene was observed. These epigenetic changes contributed to G1-phase cell cycle arrest. Although AM exhibited lower potency than trichostatin A (TSA), its HDAC inhibitory effect was more sustained over time [92,93].

Diallyl disulfide (DADS) also exhibits epigenetic activity associated with the modulation of histone acetylation and HDAC activity. In PC-3 prostate cancer cells, DADS induced apoptosis accompanied by increased histone acetylation after 24 h of incubation, suggesting either the inhibition of HDACs or activation of HATs [94]. Similarly, Druesne et al. [95] demonstrated that a 6 h incubation of Caco-2 and HT-29 colon cancer cells with 200 µM DADS increased histone H3 acetylation by 2.0-fold and 1.4-fold, respectively. An increase in histone H4 acetylation was also observed in Caco-2 cells. Analysis of nuclear extracts revealed a simultaneous reduction in HDAC activity following treatment with DADS and AM at a concentration of 200 µM. However, the inhibitory effect was markedly stronger for AM than for DADS, resulting in a 92% and 29% reduction in HDAC activity, respectively [95]. In vitro studies performed by Su et al. [96] in MGC803 gastric cancer cells showed that DADS induces hyperacetylation of histones H3 and H4 in a time- and concentration-dependent manner. Increased histone H3 acetylation was observed after 6 h, whereas increased histone H4 acetylation occurred after 12 h of incubation with 30 mg/L DADS. The strongest effect on histone H3 acetylation was observed at a concentration of 30 mg/L DADS, while maximal histone H4 acetylation was detected at 60 mg/L. The authors demonstrated that DADS exerted a stronger effect on histone H3 acetylation than on histone H4 acetylation [95]. Similar effects were observed in MCF-7 breast cancer cells, where DADS increased histone H4 acetylation and inhibited HDAC activity. In nuclear extracts from cells treated with DADS at concentrations of 10 and 100 µM, HDAC activity decreased to 85.7 ± 17.6% and 64.2 ± 12.9% of control values, respectively [96].

Diallyl trisulfide (DATS) also exhibits anticancer activity associated with HDAC inhibition and regulation of gene expression. In MDA-MB-231 triple-negative breast cancer (TNBC) cells, DATS at concentrations of 20–80 µM significantly reduced HDAC activity, whereas concentrations below 10 µM had only a minor effect. HDAC inhibition was accompanied by decreased HIF-1α mRNA expression. Overexpression of HIF-1α is associated with poor prognosis and increased metastatic potential in breast cancer; therefore, its downregulation contributed to reduced migration and the invasion of cancer cells [97]. The epigenetic activity of DATS has also been demonstrated in glioblastoma multiforme models. Using the U87MG xenograft model, Wallace et al. [98] showed that 7-day administration of DATS at doses of 10 µg/kg, 100 µg/kg, and 10 mg/kg resulted in a dose-dependent decrease in HDAC activity accompanied by increased acetylation of histones H3 and H4 [98]. The efficacy of DATS was further confirmed by Das et al. [99] in more advanced preclinical models. In SCID mice bearing orthotopic U87MG xenografts, treatment with DATS (0.1–10 mg/kg) for 14 days resulted in a significant reduction in HDAC activity and tumor volume. In a patient-derived tumor slice culture model, DATS increased histone H3 and H4 acetylation through HDAC inhibition and induced 50.1% tumor cell death. Furthermore, in an orthotopic PDX model, 14-day treatment with DATS at a dose of 10 mg/kg significantly inhibited HDAC activity, induced hyperacetylation of histones H3 and H4, and activated caspase-3. These molecular changes were accompanied by inhibition of tumor growth and prolonged animal survival, without detectable toxic effects in the central nervous system or liver [99].

Compared with fresh garlic, aged garlic extract (AGE) exhibits stronger anticancer activity, which is associated with a higher content of stable organosulfur compounds, particularly S-allylmercaptocysteine (SAMC). SAMC is a non-volatile compound formed through the reaction of allicin with L-cysteine at a pH of approximately 6 [100]. SAMC has been shown to induce histone hyperacetylation in DS19 murine erythroleukemia cells, Caco-2 colon cancer cells, and T-47D breast cancer cells. However, the mechanism underlying this effect has not been fully elucidated, and it has been suggested that it may result from the metabolism of SAMC to allyl mercaptan (AM), a previously described direct HDAC inhibitor [84].

Although AM is reported in the literature as a direct HDAC inhibitor, numerous garlic-derived organosulfur compounds, including DADS, DATS, and SAMC, induce effects characteristic of HDAC inhibition, such as histone hyperacetylation, cell cycle arrest, and apoptosis induction. These findings suggest that garlic organosulfur compounds may modulate epigenetic processes not only through direct effects on HDAC activity but also through interactions with other chromatin-regulating mechanisms, ultimately leading to biological effects similar to those observed for classical HDAC inhibitors [101]. However, despite promising results from preclinical studies, it remains unclear whether consumption of garlic or garlic-derived compounds can result in AM concentrations in human tissues sufficient to effectively inhibit HDAC activity [92]. Moreover, recent studies indicate that the anticancer effects of HDAC inhibitors are not always mediated exclusively by the inhibition of HDAC enzymatic activity [102]. Therefore, the modulation of histone acetylation may represent one component of the complex anticancer mechanism of garlic-derived organosulfur compounds, which also influence multiple other cellular pathways.

### 3.3. Formononetin (FMNT)

**Figure 6:**
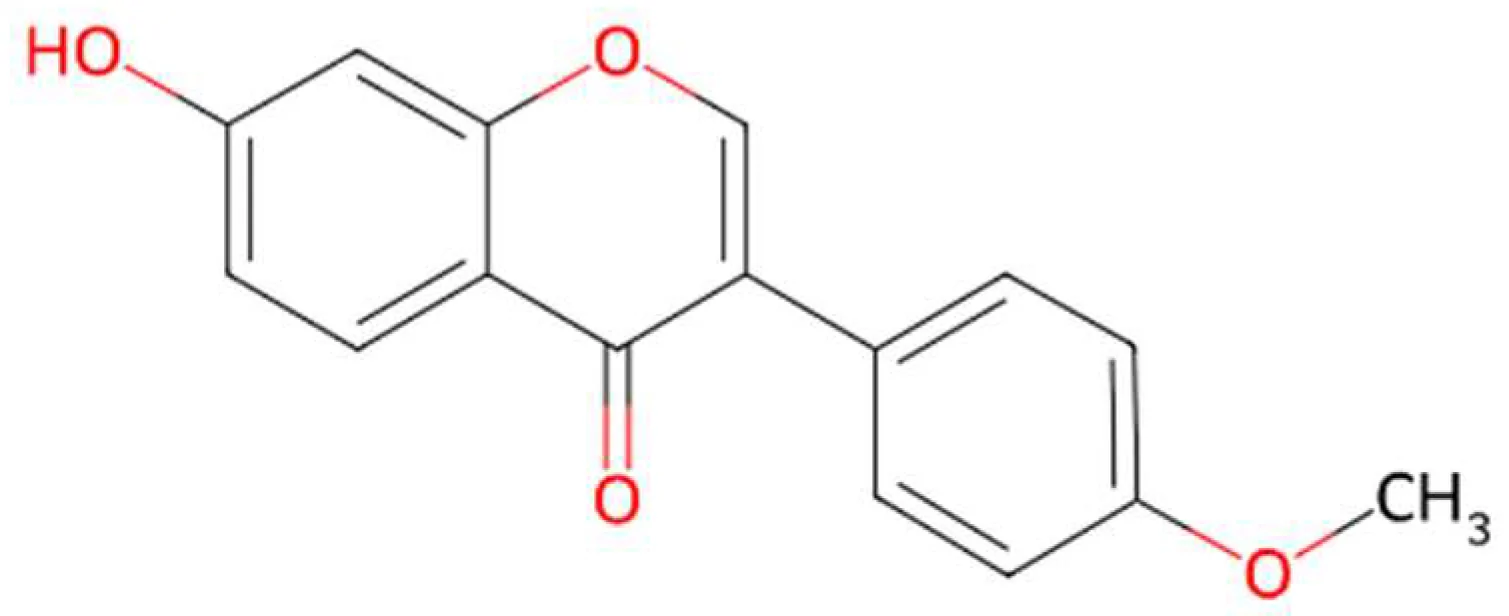
The chemical structure of formononetin.

Anticancer activity has been demonstrated for a broad range of naturally occurring compounds belonging to diverse chemical classes, including isoflavones. One representative compound is formononetin (FMNT; C_16_H_12_O_4_), also known as 7-hydroxy-4′-methoxyisoflavone, with a molecular weight of 268 g/mol (Figure 6) [103].

FMNT is a naturally occurring isoflavone that is widely distributed in plants, where it functions as a secondary metabolite. Owing to its structural similarity to endogenous estrogens and its ability to interact with estrogen receptors, FMNT is classified as a phytoestrogen. Accumulating evidence indicates that this compound exerts a broad spectrum of biological and pharmacological activities, including anticancer, antioxidant, and anti-inflammatory effects [104]. Considerable attention has been directed toward the anticancer potential of FMNT. Previous experimental studies have demonstrated that FMNT can suppress cancer cell proliferation and induce apoptotic cell death. These effects have been reported in several malignancies, including cervical, colorectal, gastric, breast, and glioma-derived cancers. Moreover, experimental evidence suggests that FMNT may attenuate tumor growth and progression in vivo through the modulation of multiple intracellular signaling pathways involved in cell survival, proliferation, apoptosis, migration, and invasion. Among the molecular mechanisms potentially underlying its antitumor activity, increasing attention has been directed toward its ability to modulate the epithelial–mesenchymal transition (EMT) [105]. EMT is a dynamic and reversible cellular plasticity program during which epithelial cells progressively lose their characteristic epithelial phenotype and acquire molecular and morphological features associated with mesenchymal cells. Under physiological conditions, EMT contributes to fundamental processes such as embryogenesis, tissue remodeling, and wound repair. However, pathological activation of EMT is closely associated with tumor initiation, malignant progression, local invasion, metastatic dissemination, and resistance to anticancer therapy. The EMT program is characterized by profound alterations in cell adhesion, polarity, cytoskeletal organization, and extracellular matrix interactions, accompanied by changes in the expression of key epithelial and mesenchymal markers [106]. Studies conducted in glioma cells have shown that doxorubicin can induce EMT, whereas the simultaneous administration of FMNT inhibits this process and may even reverse it. These findings suggest that FMNT may counteract the phenotypic changes induced by doxorubicin and thereby limit processes associated with glioma progression. The mechanism underlying this effect may be related to the regulation of the activity of enzymes involved in the control of gene expression, particularly HDACs [105]. Within this context, HDAC5 has attracted particular interest. HDAC5 belongs to the class IIa HDAC family and has been reported to be dysregulated in several human malignancies. Increasing evidence indicates that aberrant HDAC5 expression or activity may influence fundamental processes associated with tumor biology, including cell proliferation, cell-cycle progression, apoptosis, migration, invasion, and therapeutic response. Through its effects on transcriptional regulation, HDAC5 may contribute to the expression of genes involved in malignant transformation and tumor progression [107]. Importantly, experimental depletion of HDAC5 using small interfering RNA (siRNA) has been shown to significantly alter the biological behavior of glioma cells. These findings support the hypothesis that HDAC5 participates in the molecular mechanisms underlying doxorubicin-induced EMT. In parallel, the ability of FMNT to inhibit, and potentially reverse, EMT-associated changes raises the possibility that its effects are mediated, at least in part, through the modulation of HDAC5-dependent signaling and transcriptional pathways. Such regulation could subsequently affect the expression of genes governing epithelial and mesenchymal phenotypes, thereby reducing cellular characteristics associated with tumor progression [105].

Taken together, the available evidence suggests that the anticancer activity of FMNT in glioma may be mediated, at least partially, through the modulation of HDAC5-associated molecular mechanisms. By influencing HDAC5 activity or expression and consequently altering the transcriptional programs involved in EMT, FMNT may attenuate the acquisition of mesenchymal characteristics and thereby suppress cellular processes associated with glioma progression, including enhanced migratory and invasive capacity. Nevertheless, the precise molecular relationship between FMNT, HDAC5, and EMT remains to be fully elucidated, and further mechanistic studies are warranted to establish whether HDAC5 represents a direct molecular target of FMNT or rather a component of a broader regulatory network [105,108].

### 3.4. Plumbagin (PLB)

**Figure 7:**
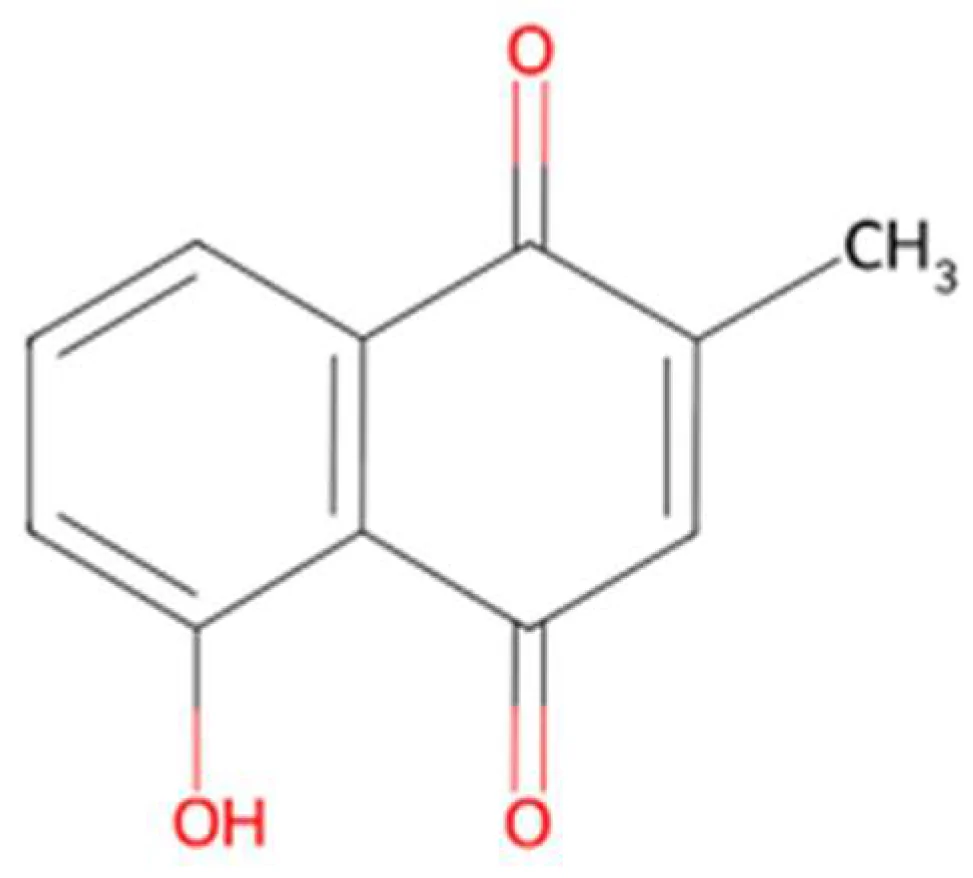
The chemical structure of plumbagin.

In recent years, increasing evidence has highlighted the considerable pharmacological potential of both natural and synthetic naphthoquinones. Plumbagin (PLB; 5-hydroxy-2-methyl-1,4-naphthoquinone) is a naturally occurring naphthoquinone isolated from the roots of the medicinal plant *Plumbago zeylanica* L. (Figure 7) [109,110].

Numerous studies have demonstrated that PLB exerts anticancer effects and may have potential applications in the treatment of various types of cancer, including breast cancer, brain tumors, lung cancer, and hepatocellular carcinoma. In addition, PLB has been reported to possess antioxidant and anti-inflammatory properties, suggesting that this compound may also have potential applications in research on neurodegenerative and cardiovascular diseases [109,110].

Interest has been directed toward the potential relationship between the effects of PLB and sirtuin (SIRT) activity. Sirtuins belong to class III histone deacetylases and are NAD^+^-dependent enzymes involved in the regulation of numerous cellular processes, including metabolism, gene expression, stress responses, aging, and nervous system function. In mammals, seven sirtuins (SIRT1-SIRT7), have been identified. Among the members of this family, SIRT1 is the best characterized, whereas SIRT2 plays an important role in the regulation of the cell cycle and processes associated with cell division [111]. SIRT2 exhibits cell cycle-dependent subcellular localization. During interphase, it is predominantly cytoplasmic, whereas during mitosis, it is associated with cellular structures involved in the regulation and execution of mitotic progression. Given the established involvement of SIRT2 in mitotic spindle organization and function, elucidating whether PLB modulates the subcellular distribution and functional activity of SIRT2 is of scientific interest [112].

To investigate the potential relationship between PLB, sirtuins, and mitotic spindle function, the effect of PLB on SIRT2 localization was assessed. First, the presence of SIRT2 within the mitotic spindle was examined by immunofluorescence. Subsequently, SIRT2 localization at the centrosomes of PLB-treated HeLa cells was analyzed and compared with untreated control cells [112]. Neural precursor cell expressed that developmentally downregulated 1 (NEDD1) was used as a centrosomal marker [113]. Comparison of NEDD1 and SIRT2 fluorescence signals revealed that, in control cells, SIRT2 was already detectable at the centrosomes during the G2 phase. Centrosomes represent the principal microtubule-organizing centers and are essential for proper mitotic spindle assembly. During prophase, identified by chromosome condensation and centrosome separation, the further accumulation of SIRT2 at the centrosomes was observed. Subsequently, during prometaphase, SIRT2 accumulated within the mitotic spindle. A distinct pattern was observed following PLB treatment. The NEDD1 signal was markedly reduced compared with control cells, particularly during G2 and prometaphase, potentially indicating impaired centrosome maturation or organization. Notably, following PLB exposure, the SIRT2 signal at the centrosomes during the G2/M transition was barely detectable. These findings indicate that PLB affects not only mitotic progression but also the proper centrosomal localization of SIRT2. Further analyses demonstrated that PLB disrupts mitotic spindle assembly and delays cell division. In addition, PLB induced pronounced protein hyperacetylation. The analysis of α-tubulin acetylation provided further evidence that PLB inhibits sirtuin activity in vivo, including SIRT2. The inhibition of SIRT2 activity may result in increased acetylation of its protein substrates. Consistent with this, PLB induced the hyperacetylation of proteins associated with the mitotic apparatus. Consequently, impaired sirtuin activity may compromise microtubule organization, mitotic spindle assembly, and mitotic progression. These findings suggest that PLB exerts its cellular effects through interconnected mechanisms involving sirtuin inhibition, altered protein acetylation, and the disruption of mitotic machinery [112].

In summary, the presented findings indicate a significant association between the effects of PLB and the sirtuin-dependent pathway, particularly SIRT2. PLB may inhibit SIRT2 activity, resulting in protein hyperacetylation, which may contribute to aberrant mitotic spindle assembly, delayed mitotic progression, and impaired cell proliferation. Given the involvement of SIRT2 in the regulation of numerous proteins associated with cellular and pathological processes, its inhibition by PLB may represent one of the mechanisms underlying the anticancer activity of this compound and may have potential therapeutic relevance [112].

### 3.5. Anacardic Acid (AA)

**Figure 8:**
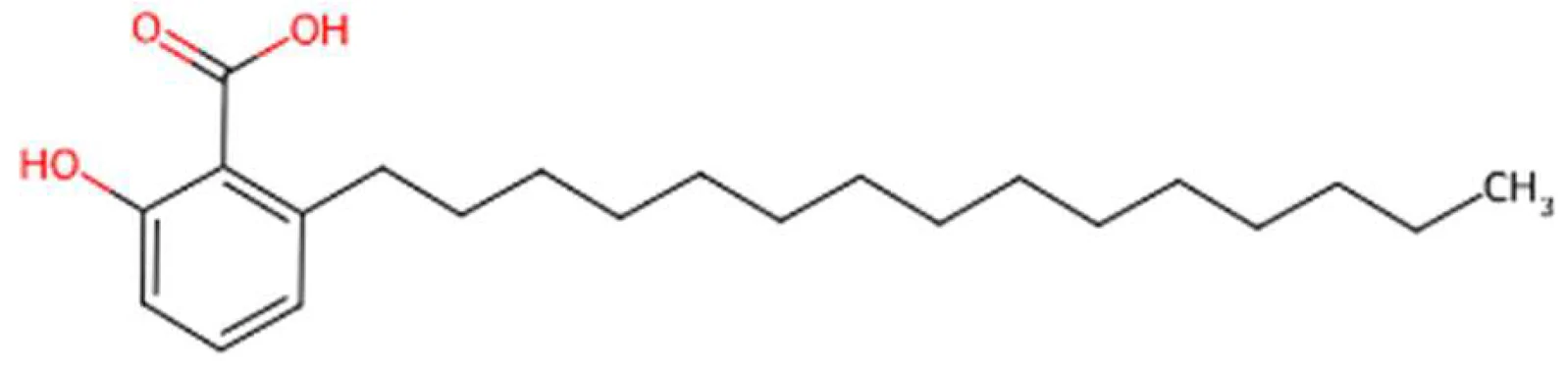
The chemical structure of anacardic acid.

Anacardic acids (AAs) refer to a group of 2-hydroxy-6-alkylbenzoic acid derivatives and a major constituent of cashew nutshell liquid (CNSL). The main components of naturally occurring AA comprise salicylic acid derivatives with a 15-carbon side chain differing in the degree of unsaturation (Figure 8) [114].

AA has attracted considerable attention because of its broad range of biological activities, including antimicrobial, antioxidant, anti-inflammatory, and antitumor effects. Among its molecular targets, HATs have been particularly extensively investigated because of their central role in the regulation of chromatin structure, gene transcription, and DNA damage responses [114,115].

AA was initially characterized as a potent noncompetitive inhibitor of HAT activity, with inhibitory effects against both p300 and p300/CBP-associated factors (PCAFs). In vitro, AA inhibited p300 and PCAF with IC_50_ values of approximately 8.5 and 5 μmol/L, respectively [115]. These findings established AA as one of the first naturally occurring small molecules capable of pharmacologically modulating HAT activity and stimulated subsequent efforts to develop AA-derived HAT inhibitors and activators. However, subsequent studies demonstrated that the reported potency of AA can vary substantially depending on enzyme preparation and experimental assay. For example, independent studies reported the considerably weaker inhibition of p300, PCAF, and Tip60 than that observed in the original study, indicating that the HAT-inhibitory activity of AA should be interpreted in an assay-dependent context [116,117].

The relatively limited selectivity of AA toward individual HATs prompted efforts to modify its chemical scaffold while preserving its characteristic salicylate structure. Initial structural studies focused on modifications of the phenolic ring and the introduction of substituted anilide groups. These modifications generated derivatives with markedly different effects on individual HATs. One of the most notable compounds was N-(4-chloro-3-trifluoromethylphenyl)-2-ethoxy-6-pentadecylbenzamide (CTPB), an amide derivative of AA that activated p300 HAT activity while having little effect on PCAF [115]. The stimulatory effect of CTPB was also reflected at the transcriptional level and was associated with increased histone acetylation within promoter-proximal regions. These observations demonstrated that relatively small structural changes within the AA scaffold can substantially alter its activity toward individual HATs and suggested that AA could serve as a useful starting point for the development of selective HAT modulators [115].

The observation that AA-derived compounds can activate rather than inhibit HAT activity was further supported by the identification of pentadecylidenemalonate (SPV106), a simplified structural analog of AA. Pentadecylidenemalonate displayed an unusual mixed activity profile, inhibiting p300/CBP while simultaneously enhancing PCAF HAT activity. The compound also exhibited apoptotic activity in U937 leukemia cells, suggesting that selective modulation of individual HATs may have biological and potentially therapeutic consequences [118]. Subsequent mechanistic studies further characterized pentadecylidenemalonate as HAT activator/inhibitor and investigated its interactions with PCAF and CBP, providing a structural basis for the further optimization of this class of compounds [119]. These observations are particularly important because they demonstrate that the AA scaffold does not necessarily have to be developed exclusively toward HAT inhibition; it can also provide a starting point for selective HAT activation or bidirectional modulation.

Further studies focused specifically on improving the inhibitory activity of AA derivatives toward PCAF. A molecular model describing the binding of AA within the PCAF active site was developed and used as a structural basis for the rational design of novel derivatives. Attention was given to modifications of the 6-alkyl chain, which was replaced with different substituents to improve inhibitory potency and physicochemical properties. Ten AA derivatives were subsequently synthesized and evaluated using recombinant PCAF HAT, while their effects on histone acetylation were additionally investigated in HepG2 cells. One derivative showed a twofold improvement in PCAF HAT inhibition and a twofold improvement in inhibition of histone acetylation in HepG2 cells, demonstrating that the structural optimization of the AA scaffold can enhance both biochemical and cellular activity [120].

An additional series of AA analogs was developed by introducing a second aromatic ring, modifying the length and position of the alkyl chain, and introducing different substituents. A total of 28 compounds were evaluated for their HAT-inhibitory properties and effects on cancer cell growth. Although the inhibitory activity varied considerably among the derivatives, none was more potent than AA against total HAT activity under the experimental conditions used. The conversion of ethyl esters to the corresponding carboxylic acids was the most effective structural modification and increased HAT-inhibitory potency by up to 10-fold for selected compounds. Several derivatives also inhibited p300 activity in MCF7 cells and reduced histone H4 acetylation, providing evidence that structural changes in the AA scaffold can translate into cellular effects [121].

The structural versatility of AA was further demonstrated by the development of 6-alkylsalicylate derivatives targeting the MYST-family acetyltransferase Tip60. Several AA analogs showed selective inhibition of recombinant Tip60 compared with PCAF and p300. One of the most active compounds, later referred to as MG149, inhibited Tip60 with an IC_50_ of approximately 74 μM and also inhibited MOF with an IC_50_ of approximately 47 μM. Kinetic studies indicated that Tip60 inhibition was essentially competitive with acetyl-CoA and noncompetitive with the histone substrate, suggesting interaction with the acetyl-CoA-binding region of the enzyme [122]. These findings demonstrated that modification of the 6-alkyl chain can shift the activity profile of AA from relatively broad HAT inhibition toward preferential inhibition of MYST-family acetyltransferases.

The development of AA-derived HAT modulators was subsequently extended to leukemia models. MG153, another AA derivative optimized for PCAF inhibition, was investigated in BCR-ABL-expressing cells, which exhibited increased PCAF expression and resistance to apoptosis. The pharmacological inhibition of PCAF with MG153 decreased cell proliferation and induced apoptosis, accompanied by loss of mitochondrial membrane potential and DNA fragmentation. Importantly, BCR-ABL-expressing cells were more sensitive to PCAF inhibition than parental cells, and inhibition of PCAF reduced resistance to DNA damage-induced cell death. These findings suggested that PCAF inhibition may represent a potential strategy for targeting BCR-ABL-positive leukemia cells and for enhancing the effects of DNA-damaging agents [123].

Further structural optimization of pentadecylidenemalonate demonstrated that changes in the carbonyl functionality can produce markedly different activity profiles toward KAT2B/PCAF and KAT3B/p300. A series of 2-alkylidene-1,3-dicarbonyl derivatives was therefore synthesized and evaluated, leading to compounds displaying selective PCAF activation, broad KAT inhibition, or mixed PCAF activation/p300 inhibition. These findings further emphasized the functional plasticity of the AA-derived scaffold and demonstrated that subtle modifications of the dicarbonyl moiety can determine whether a compound acts as an acetyltransferase inhibitor or activator [124].

The inhibitory activity of AA is not restricted to p300 and PCAF. AA was also shown to inhibit the MYST-family acetyltransferase Tip60 and to interfere with the Tip60-dependent activation of the ATM and DNA-PKcs protein kinases following DNA damage. Treatment of human tumor cells with AA increased their sensitivity to ionizing radiation, suggesting that interference with Tip60-dependent DNA damage responses may contribute to the radiosensitizing activity of AA [125]. These findings provided an additional link between HAT inhibition and cancer therapy, particularly in the context of DNA damage and radiotherapy.

Consistent with these observations, AA was subsequently reported to sensitize prostate cancer cells to radiation therapy through the regulation of H2AX expression. AA treatment altered H2AX and phosphorylated H2AX levels and increased the response of prostate cancer cells to ionizing radiation [126]. Together, these studies indicate that the effects of AA on HAT-dependent processes may extend beyond transcriptional regulation and include the modulation of cellular responses to DNA damage.

The biological relevance of the AA scaffold has also been demonstrated through more recent studies of naturally occurring AA derivatives. In 2024, Hatakeyama et al. [127] isolated nine AA derivatives from the fungus Tyromyces fissilis and evaluated them for PCAF inhibitory activity. Two derivatives, AAd7 and AAd11, inhibited PCAF at concentrations of 50 and 100 μM, respectively. Docking simulations suggested that AA, AAd7, and AAd11 may occupy the same active-site pocket of PCAF. Interestingly, relatively small differences in functional groups within the alkyl side chain substantially affected acetyltransferase inhibitory activity, further supporting the importance of side-chain structure for the activity of AA-derived HAT modulators [127]. These recent findings indicate that the AA scaffold continues to provide a useful starting point for the identification of structurally diverse HAT inhibitors.

Beyond its direct effects on HAT activity, AA has been extensively investigated for its anticancer properties. One particularly relevant mechanism involves synthetic lethality in PTEN-deficient colorectal cancer (CRC) cells. A study using PTEN-isogenic CRC models identified p300/CBP HAT inhibition as a vulnerability of PTEN-deficient cells [128]. Inhibition of p300/CBP HAT activity reduced histone acetylation at promoter regions and suppressed the transcription of Hsp70 family proteins. Reduced Hsp70 expression impaired the formation of the AKT1-Hsp70 complex, resulting in AKT1 destabilization and depletion of AKT1 protein. Because PTEN-deficient cells exhibit constitutive activation of PI3K-AKT signaling and increased dependence on AKT-mediated survival, disruption of AKT1 stability was proposed as an important mechanism underlying the selective sensitivity of PTEN-deficient CRC cells to HAT inhibition [128]. These findings provide a mechanistic link between epigenetic regulation, oncogenic signaling, and synthetic lethality.

AA has also demonstrated anticancer activity in breast and prostate cancer models. In breast cancer cells, AA was shown to inhibit estrogen receptor α (ERα) binding to DNA and reduce ERα-dependent target gene transcription and cell proliferation [129]. These findings suggest that modulation of protein acetylation and transcriptional regulation by AA may influence hormone-dependent signaling pathways. In prostate cancer cells, AA was shown to induce apoptosis through mechanisms involving endoplasmic reticulum (ER) stress, autophagy, and the modulation of the DAPK3/Akt signaling pathway [126]. These observations further support the involvement of multiple interconnected signaling pathways in the cellular response to AA.

AA has also been investigated in combination with conventional anticancer therapies. In pancreatic cancer cells, AA inhibited tumor cell growth and potentiated the effects of chemotherapeutic treatment through mechanisms involving the Chmp1A-ATM-p53 signaling pathway [130]. These findings suggest that AA may have potential as a chemosensitizing compound, although the contribution of HAT inhibition to these effects remains to be fully established.

The antiproliferative activity of AA has also been demonstrated in rhabdomyosarcoma (RMS) cells. AA and the natural HAT inhibitor garcinol (GA) reduced cell proliferation, viability, and clonogenic potential in a concentration-dependent manner. GA was more potent than AA in this model, with reported IC_50_ values of approximately 15 and 54 μM, respectively [131]. Both compounds also affected cell-cycle progression, with G2/M arrest being particularly evident in RMS cells. Treatment altered the expression of genes associated with ER stress and apoptosis, including DDIT3, DDIT4, SESN2, and TRIB3 [131]. These findings suggest that ER-stress-related signaling may contribute to the antiproliferative and pro-apoptotic effects of AA and GA in RMS cells.

Importantly, the biological effects of AA are not uniformly antiproliferative across all cellular models. In contrast to the growth-inhibitory effects reported in several malignancies, AA was shown to enhance the proliferation of human ovarian cancer cells. AA treatment was also associated with increased migration and invasion, increased PI3K and VEGF expression, and reduced caspase-3 expression [132]. These findings indicate that the cellular response to AA may be strongly dependent on tissue type, molecular background, concentration, and experimental context. Such observations should be considered when assessing the potential therapeutic application of AA.

Despite the extensive experimental evidence supporting the biological activity of AA, several limitations should be considered when interpreting its potential as a pharmacological HAT inhibitor. First, the reported potency and selectivity of AA toward individual HATs are not entirely consistent between experimental systems. Independent studies have reported substantially different inhibitory activities toward p300, PCAF, and Tip60, suggesting that assay conditions, enzyme preparation, substrate composition, and compound properties may influence the observed activity [116,117]. In addition, AA is highly lipophilic and has limited aqueous solubility, which may affect its cellular availability and complicate the interpretation of concentration-dependent cellular effects [116,121]. AA also interacts with molecular targets beyond HATs, and therefore cellular responses cannot necessarily be attributed exclusively to inhibition of p300, PCAF, or Tip60 [116]. These limitations have stimulated the development of structurally modified AA derivatives with improved selectivity, potency, and physicochemical properties.

Overall, AA represents a versatile natural-product scaffold capable of modulating several HAT families and influencing multiple cancer-associated signaling pathways. Its biological activity includes inhibition of p300/PCAF and Tip60, modulation of HAT-dependent transcription, interference with DNA damage responses, induction of apoptosis and ER stress, modulation of AKT signaling, and enhancement of responses to selected chemotherapeutic and radiotherapeutic treatments. Importantly, structural modification of AA can generate compounds with substantially different HAT selectivity profiles, including selective PCAF inhibitors, Tip60/MOF inhibitors, p300 activators, and mixed HAT activator/inhibitors. The continued identification of AA derivatives, including naturally occurring compounds reported as recently as 2024, indicates that this scaffold remains a useful starting point for the development of novel HAT-targeting compounds [127]. However, the variable potency, limited selectivity, physicochemical limitations, and context-dependent cellular effects of AA indicate that further structural optimization and mechanistic studies are required before AA-derived compounds can be considered as clinically relevant HAT-targeting agents.

## 4. Challenges in Clinical Translation

Despite encouraging preclinical findings, the clinical translation of PTL, FMNT, PLB, AA, and garlic-derived organosulfur compounds remain hindered by interrelated pharmacokinetic, toxicological, and pharmaceutical limitations. Poor aqueous solubility limited systemic bioavailability and metabolic instability; and rapid elimination may compromise their ability to achieve and sustain pharmacologically relevant concentrations within tumor tissues. These limitations have been reported for PTL and PLB, whereas FMNT undergoes extensive biotransformation, and the cellular permeability of AA remains an important consideration [117,133,134,135,136]. Similarly, the chemical instability and rapid transformation of allicin into other organosulfur metabolites complicate the characterization of its systemic exposure and the identification of the molecular species responsible for its biological activity [137].

Beyond these pharmacokinetic constraints, the mechanistic specificity of the reported epigenetic effects warrants critical consideration. Although these phytochemicals have been associated with alterations in HAT/HDAC-related pathways, changes in histone acetylation alone are insufficient to establish direct enzymatic modulation. Rather, their pleiotropic effects on redox homeostasis, inflammatory signaling, and other intracellular regulatory networks may contribute to the observed anticancer responses, while simultaneously increasing the potential for off-target effects. Accordingly, definitive elucidation of their mechanisms of action requires complementary biochemical and cellular assays, direct target-engagement studies, and the systematic evaluation of selectivity [58,135].

In line with these translational limitations, clinical evidence of the therapeutic efficacy of the phytochemicals discussed remains limited. A ClinicalTrials.gov study NCT00455416, entitled Dietary Intervention in Stage III/IV Follicular Lymphoma, was designed to evaluate the effects of a multimodal dietary intervention incorporating garlic extract containing allicin, omega-3 fatty acids, selenium, pomegranate juice, grape juice, and green tea on tumor-cell apoptosis and proliferation, as well as the composition of the tumor-associated immune-cell infiltrate [138]. Nevertheless, the concomitant administration of multiple dietary components precludes the unequivocal attribution of any observed biological effects to allicin or other individual constituents. Moreover, the study registration and its specified outcome measures do not, in themselves, substantiate clinical efficacy or establish a mechanistic association between the intervention and HAT/HDAC modulation. Accordingly, this study illustrates the preliminary clinical investigation of dietary bioactive compounds in oncology, while underscoring the need for controlled compound-specific trials incorporating appropriate pharmacokinetic and pharmacodynamic endpoints.

In this context, advanced drug-delivery technologies represent a promising strategy for addressing selected pharmaceutical barriers. Liposomes, polymeric nanoparticles, micelles, and lipid-based carriers may enhance solubility, protect chemically unstable compounds from degradation, and facilitate controlled drug release. Preclinical investigations involving PTL, FMNT, PLB, AA support further exploration of these approaches [133,136,137,139]. Nevertheless, their translational potential must be substantiated through rigorous assessment of biodistribution, tumor penetration, release kinetics, immunogenicity, and formulation-associated toxicity. Ultimately, progression toward clinical application will require standardized compound preparations, comprehensive pharmacokinetic and pharmacodynamic characterization, confirmation of target engagement at clinically achievable exposures, and appropriately designed clinical trials.

## 5. Discussion and Future Opportunities

Plant-derived phytochemicals represent a diverse group of compounds with potential to modulate HAT- and HDAC-associated pathways in cancer. However, their mechanisms of action differ considerably. The compounds discussed in the review—PTL, garlic-derived organosulfur compounds, FMNT, PLB, and AA—illustrate distinct mechanisms ranging from direct enzymatic inhibition to indirect regulation of enzyme abundance, histone acetylation, and downstream signaling. Compared with extensively investigated natural epigenetic modulators, including curcumin, sulforaphane, epigallocatechin-3-gallate (EGCG), resveratrol, and genistein, the phytochemicals discussed in this review represent less extensively characterized candidates for HAT/HDAC modulation. Established compounds have been widely studied for their effects on histone-modifying enzymes, chromatin organization, and cancer-related signaling pathways, providing a broader evidence base for their epigenetic activities. In contrast, PTL, garlic-derived organosulfur compounds, FMNT, PLB, and AA exhibit distinct mechanistic profiles that warrant further investigation.

Among the selected compounds, AA has some of the strongest biochemical evidence for direct HAT modulation, particularly inhibition of p300 and PCAF, with initially reported IC_50_ values of approximately 8.5 and 5 μM, respectively [115]. However, subsequent studies have demonstrated variable inhibitory potency and substantial differences in target selectivity depending on the experimental conditions and chemical structure [116,117]. AA derivatives can also exhibit distinct or opposing effects on HAT activity, highlighting the potential of this scaffold for developing more selective epigenetic modulators [122,123,124]. Despite promising findings in several cancer models, limited aqueous solubility, inconsistent potency, and context-dependent biological effects restrict its translational potential. The observation that AA can promote proliferation and invasion in certain ovarian cancer models further emphasizes the importance of tumor-specific evaluation [129].

Garlic-derived organosulfur compounds also demonstrate promising HDAC-associated activity, although the strength of evidence varies between individual compounds. AM is comparatively well-characterized as a direct HDAC inhibitor, with a reported IC_50_ of approximately 20 μM in a cell-free assay [92]. DADS and DATS have been associated with reduced HDAC activity, increased histone H3/H4 acetylation, and antitumor effects in preclinical models [94,95,96,97,98,99]. DATS has demonstrated activity in experimental glioblastoma models, including xenograft and patient-derived tumor systems [98,99]. Nevertheless, increased histone acetylation does not independently establish direct enzymatic inhibition, and the mechanisms underlying the effects of allicin and SAMC remain incompletely characterized [84]. The rapid degradation of allicin under physiological conditions represents an additional pharmacological limitation [85]. Accordingly, AM currently provides stronger evidence for direct HDAC inhibition than several other garlic-derived compounds, whereas the precise mechanisms of DADS, DATS, and related metabolites require further investigation.

PTL is distinguished by its pleiotropic anticancer activity and potential to enhance the effects of established HDIs. Its reported mechanisms include suppression of NF-κB signaling, induction of oxidative stress and apoptosis, and modulation of HDAC1 protein abundance [63,64,65,67,68]. Importantly, ATM- and proteasome-dependent HDAC1 depletion supports an indirect mechanism rather than demonstrating direct inhibition of the HDAC1 catalytic site [68]. PTL enhanced the effects of vorinostat and panobinostat in AML models, supporting its potential as a combination partner [63]. However, inadequate systemic exposure remains a major limitation: in a phase I study, plasma PTL concentrations following oral feverfew administration remained below the assay detection limit [67]. Therefore, improved formulations and more bioavailable derivatives require further evaluation before its clinical potential can be established.

FMNT and PLB represent less fully characterized candidates for epigenetic modulation. In glioma models, FMNT has been associated with the regulation of HDAC5-related pathways and attenuation of doxorubicin-induced EMT [105]. However, direct interaction with or inhibition of HDAC5 has not been conclusively demonstrated. PLB has been associated with altered SIRT2 localization, increased protein acetylation, and disruption of mitotic spindle organization [112]. Nevertheless, these observations do not establish direct SIRT2 inhibition. Thus, both compounds are more appropriately described as candidate modulators of HDAC5- and sirtuin-associated processes, respectively, pending further biochemical and target-engagement studies.

Collectively, these findings demonstrate that the selected phytochemicals differ in mechanistic evidence and potential therapeutic applications. AA and AM have comparatively clear evidence supporting direct HAT or HDAC inhibition, respectively, whereas DADS and DATS exhibit promising HDAC-associated effects that require further mechanistic clarification. PTL may be particularly relevant to combination therapy, while FMNT and PLB require additional investigation to establish their molecular targets. Although the pleiotropic activity of these compounds may be advantageous in tumors characterized by interconnected survival, inflammatory, and epigenetic pathways, it may also increase off-target effects and complicate the attribution of anticancer activity to a specific epigenetic mechanism.

Several pharmacological barriers must be addressed before these compounds can be considered clinically relevant anticancer adjuvants. Bioavailability and metabolic stability are particularly important, as illustrated by the rapid degradation of allicin, the inadequate systemic exposure reported for PTL, and the limited aqueous solubility of AA [67,85,116,121]. Furthermore, toxicity and target selectivity remain insufficiently characterized for many of the compounds. Their effects on multiple molecular pathways may influence both tumor cells and normal tissues, making it essential to establish therapeutic windows and evaluate tissue-specific responses. For FMNT, estrogen receptor-related activity also warrants consideration, particularly in hormone-sensitive malignancies [104,105].

The translation of preclinical findings into clinical treatments presents additional challenges. Enzyme inhibition in cell-free assays, changes in histone acetylation, and tumor suppression in experimental models do not necessarily predict clinical efficacy. Differences in drug exposure, metabolism, tumor heterogeneity, and biological context may substantially affect therapeutic outcomes. Moreover, enhanced cytotoxicity in combination studies does not necessarily demonstrate pharmacological synergy or prove that the observed effect is mediated by HAT/HDAC modulation. Future studies should therefore prioritize target-engagement assays, isoform-selective enzymology, genetic validation, pharmacokinetic and pharmacodynamic characterization, and systematic toxicity assessment. Structure–activity optimization and biomarker-guided combination strategies may further improve selectivity and identify tumor subtypes most likely to respond.

## 6. Conclusions

Plant-derived compounds such as PTL, garlic organosulfur metabolites [140], FMNT, PLB, and AA constitute a structurally diverse reservoir of HAT/HDAC modulators whose anticancer effects may arise from direct enzymatic regulation as well as the indirect remodeling of interconnected epigenetic and signaling networks. Their greatest translational potential may lie not as standalone anticancer agents, but as molecularly defined adjuvants capable of sensitizing tumors to established epigenetic, cytotoxic, or DNA-damaging therapies. Rigorous target validation, pharmacological optimization, and biomarker-guided preclinical and clinical studies are now required to establish whether these promising natural scaffolds can be translated into selective and clinically meaningful epigenetic therapeutics.

**Table 1:** Phytochemical compounds as potential HAT and HDAC modulators. AA—Anacardic acid; AAd7—Anacardic acid derivative 7; AAd11—Anacardic acid derivative 11; AM—Allyl mercaptan; ATM—Ataxia-telangiectasia mutated protein kinase; CBP—CREB-binding protein; Caco-2—Human colorectal adenocarcinoma cell line; CTPB—N-(4-chloro-3-trifluoromethylphenyl)-2-ethoxy-6-pentadecylbenzamide; DADS—Diallyl disulfide; DATS—Diallyl trisulfide; FMNT—Formononetin; GSCs—Glioma stem-like cells; H3—Histone H3; H4—Histone H4; HAT—Histone acetyltransferase; HDAC—Histone deacetylase; HepG2—Human hepatocellular carcinoma cell line; HIF-1α—Hypoxia-inducible factor 1-alpha; HT-29—Human colorectal adenocarcinoma cell line; IC_50_—Half-maximal inhibitory concentration; KAT—Lysine acetyltransferase; MEFs—Mouse embryonic fibroblasts; NF-κB—Nuclear factor kappa B; N/A—Not applicable/not available; PCAF—p300/CBP-associated factor; p300—E1A-binding protein p300, a histone acetyltransferase; PDX—Patient-derived xenograft; PTEN—Phosphatase and tensin homolog; PTL—Parthenolide; PLB—Plumbagin; p53 WT—Wild-type p53; SAMC—S-allylmercaptocysteine; SAHA—Suberoylanilide hydroxamic acid (vorinostat); SCID—Severe combined immunodeficiency; SPV106—Synthetic anacardic acid-derived compound; Tip60—Tat-interacting protein, 60 kDa (KAT5); TPA—12-O-tetradecanoylphorbol-13-acetate; Ac-H3—Acetylated histone H3; Ac-H4—Acetylated histone H4; H3K9me2—Histone H3 dimethylated at lysine 9; H3K4me2—Histone H3 dimethylated at lysine 4; HDAC1/2/5/8—Histone deacetylas isoforms 1, 2, 5 and 8; WT—Wild type; μM—Micromolar; mg/L—Milligrams per liter; μg/kg—Micrograms per kilogram; ↑—Increase; ↓—Decrease.

Phytochemical	Botanical Source	Experimental Model	Changes in Histone Acetylation	Concentration/IC_50_	Regulation of HDAC/HAT	Clinical Status	Reference(s)
**Parthenolide** (**PTL**)	*Tanacetum parthenium* (L.)	Human ZR-75-1 breast carcinoma cells; cell-free in vitro assay.	Increased global histone H3 acetylation.	15 µM	HDAC1 protein expression decreased; no direct inhibition of isolated HDAC1 enzyme activity.	Preclinical in vitro.	[68]
		HCT-116 colorectal cancer cells (p53 WT and p53-deficient); ATM-proficient/deficient fibroblasts; mouse embryonic fibroblasts (MEFs) with selected signaling-gene deletions.	N/A	15 µM	Selective ATM-dependent proteasomal depletion of HDAC1 protein.	Preclinical in vitro.	[68]
		Cell-free HDAC activity assay; HDAC2 molecular docking (in silico).	N/A	5 and 20 µM	Inhibition of nuclear HDAC activity demonstrated experimentally; predicted binding to HDAC2 by molecular docking.	Preclinical biochemical and computational evidence.	[69]
		Murine JB6P + promotion-sensitive epidermal cells, with or without TPA stimulation; JB6P − promotion-resistant cells as controls.	No major changes in global histone H3/H4 acetylation.	5–10 µM	No significant change in global HDAC activity; HDAC1 cleavage observed.	Preclinical in vitro.	[70]
		Human U87 and A172 glioma cells; U87-derived glioma stem-like cells (GSCs).	N/A	4 µM	HDAC1 protein expression decreased; direct HDAC1 inhibition not established.	Preclinical in vitro.	[71]
		MDA-MB-231 triple-negative breast cancer cells; PTL + SAHA combination.	H3/H4 hyperacetylation induced by SAHA and maintained during combination treatment; not attributable to PTL alone.	SAHA 2 µM pretreatment (20 h) followed by PTL 10 µM + SAHA	SAHA-mediated HDAC inhibition and histone hyperacetylation; direct PTL-mediated HDAC/HAT regulation was not established.	Preclinical in vitro.	[72]
**Allicin**	*Allium sativum* L.	DS19 murine erythroleukemia cells.	Increased mono-, di-, and tri-acetylated histones after 24 h.	[µM]	Direct HDAC inhibition has not been demonstrated.	Preclinical in vitro; clinical NCT00455416 trial	[84]
**Allyl mercaptan** (**AM**)	*Allium sativum* L.	DS19 murine erythroleukemia cells, HDAC activity measured in nuclear extracts.	Increased histone acetylation	0.5 mM	HDAC activity reduced by 74%.	Preclinical in vitro.	[91]
		Nuclear extracts of Caco-2 cells.	Increased histone H3/H4 acetylation.	200 µM	HDAC activity reduced by 92%.	Preclinical in vitro.	[95]
		HT-29 colon cancer cells.	Histone H3 hyperacetylation at the *P21WAF1* promoter after 4 h.	0.5–2.0 mM	N/A	Preclinical in vitro.	[92]
		Cell-free HDAC assay.	N/A	IC_50_ = 20 µM	Clear dose-dependent HDAC inhibition; competitive inhibition of HDAC8.	Preclinical in vitro.	[92]
**Diallyl disulfide** (**DADS**)	*Allium sativum L.; Allium ursinum*	PC-3 prostate cancer cells.	Increased histone acetylation.	25 and 40 µM	Suggested HDAC inhibition or HAT activation; neither directly confirmed.	Preclinical in vitro.	[94]
		Caco-2 and HT-29 colon cancer cells.	Histone H3 and H4 acetylation increased.	200 µM	Possible contribution suggested, but HAT activation was not established.	Preclinical in vitro.	[95]
		Nuclear extracts of Caco-2 cells.	N/A	200 µM	HDAC activity was reduced by 29%.	Preclinical in vitro.	[95]
		MGC803 gastric cancer cells.	H3 and H4 hyperacetylation; H3 and H4 acetylation increased.	30 and 60 mg/L	HDAC inhibition suggested; direct mechanistic evidence not established in this study.	Preclinical in vitro.	[96]
		MCF-7 breast cancer cells; nuclear extracts.	Increased histone H4 acetylation.	10 and 100 µM	HDAC activity decreased to 85.7 and 64.2 of control, respectively.	Preclinical in vitro.	[140]
**Diallyl trisulfide** (**DATS**)	*Allium sativum L.; Allium ursinum*	MDA-MB-231 triple-negative breast cancer cells.	N/A	20–80 µM	Reduction in HDAC activity Minor effect at concentrations below 10 µM.	Preclinical in vitro.	[97]
		U87MG xenograft model.	Increased histone H3 and H4 acetylation.	7-day administration: 10 µg/kg, 100 µg/kg, and 10 mg/kg	Dose-dependent HDAC inhibition.	Preclinical; in vivo animal study.	[98]
		SCID mice with orthotopic U87MG xenografts.	N/A	0.1–10 mg/kg	Dose-dependent HDAC inhibition.	Preclinical; in vivo animal study.	[99]
		Orthotopic PDX model.	Increased histone H3 and H4 acetylation.	10 mg/kg		Preclinical; in vivo animal study.	[99]
		Patient-derived tumor-slice culture.	Increased histone H3 and H4 acetylation.	N/A		Preclinical.	[99]
**S-allylmercaptocysteine** (**SAMC**)	*Allium sativum L.*	DS19 murine erythroleukemia cells; Caco-2 colon cancer cells; T-47D breast cancer cells.	Histone hyperacetylation.	[μM]	Mechanism not fully elucidated; the effect may be mediated by metabolism to allyl mercaptan (AM).	Preclinical in vitro.	[84]
**Formononetin** (**FMNT**)	*Astragalus membranaceus*	Human U87 and U251 glioma cells.	Not directly reported.	0–200 μM	HDAC5 inhibition.	Preclinical in vitro.	[105]
**Plumbagin** (**PLB**)	*Plumbago zeylanica L.*	Human HeLa cervical cancer cells.	Increased protein acetylation.	IC_50_ not specified.	SIRT2 inhibition.	Preclinical in vitro.	[112]
**Anacardic acid** (**AA**)	*Anacardium occidentale*	Cell-free in vitro assay.	Decreased H3 and H4 acetylation.	IC_50_: 8.5 μM (p300); 5 μM (PCAF)	Noncompetitive inhibition of HATs (p300 and PCAF).	Preclinical in vitro.	[115]
		Human tumor cells exposed to ionizing radiation.	N/A	9 and 30 μM	Tip60 inhibition.	Preclinical in vitro.	[125]
		LNCaP prostate cancer cells.	N/A	12,5, 25, 50 μM	HAT-related effects investigated; exact contribution not fully described.	Preclinical in vitro.	[126]
		PTEN-isogenic colorectal cancer cells.	Decreased H4 acetylation	20–100 μM	p300/CBP HAT inhibition.	Preclinical in vitro.	[128]
		Breast cancer cells.	N/A	10–50 μM	Potential modulation of acetylation-dependent transcription.	Preclinical in vitro.	[129]
		Rhabdomyosarcoma cells	N/A	IC_50_: approximately 52 and 54 μM	HAT inhibition	Preclinical in vitro.	[131]
**CTPB**	Synthetic AA-derived compound	Cell-free in vitro assay.	Increased H3 and H4 acetylation.	25–300 μM	Activates p300 with little effect on PCAF.	Preclinical in vitro.	[115]
**SPV106**	Synthetic AA analog	Cell-free in vitro assay; U937 leukemia cells.	Increased H3 acetylation.	25 and 50 μM	Inhibits p300/CBP while activating PCAF.	Preclinical in vitro.	[118,119]
**AA-derived PCAF inhibitors**	Synthetic AA derivatives	Cell-free in vitro assay; HepG2 cells.	Decreased histone acetylation.	30–60 μM	Enhanced PCAF inhibition.	Preclinical in vitro.	[120]
**AA analogs with modified aromatic rings and alkyl chains**	Synthetic AA derivatives	Cell-free in vitro assays; MCF7 breast cancer cells.	Decreased histone H4 acetylation for selected compounds.	6–90 μM	Inhibition of total HAT activity and p300.	Preclinical in vitro.	[121]
**Compound 20**	Synthetic 6-alkylsalicylate derivative	Cell-free in vitro assays; HeLa cervical cancer cells.	Decreased H3 and H4 acetylation.	IC_50_: 74 μM (Tip60); 47 μM (MOF)	Inhibition of MYST-family acetyltransferases.	Preclinical in vitro.	[122]
**MG153**	Synthetic AA derivative	BCR-ABL-expressing cells and parental leukemia cells.	Decreased histone acetylation.	Not specified in the available abstract.	Inhibition of PCAF.	Preclinical in vitro.	[123]
**2-Alkylidene-1,3-dicarbonyl derivatives**	Synthetic SPV106-related compounds	Cell-free in vitro assays; HeLa cervical cancer cells.	Mixed histone modulation.	100 μM	Selective PCAF activation, KAT inhibition, PCAF activation combined with p30 inhibition.	Preclinical in vitro.	[124]
**AAd7 and AAd11 anacardic acid** (**AA**) **derivatives**	*Tyromyces fissilis*	Cell-free in vitro assays; molecular docking.	Decreased histone acetylation.	50 μM (AAd7); 100 μM (AAd11)	PCAF inhibition.	Preclinical in vitro.	[127]

## Data Availability

No new data were created or analyzed in this study. Data sharing is not applicable to this article.

## Abbreviations

AA	Anacardic acid
AGE	Aged garlic extract
AM	Allyl mercaptan
AML	Acute myeloid leukemia
CNSL	Cashew nutshell liquid
CTPB	N-(4-chloro-3-trifluoromethylphenyl)-2-ethoxy-6-pentadecylbenzamide
CRC	Colorectal cancer
DADS	Diallyl disulfide
DATS	Diallyl trisulfide
DAS	Diallyl sulfide
DMAPT	Dimethylaminoparthenolide
DNA	Deoxyribonucleic acid
EMT	Epithelial–mesenchymal transition
ER	Estrogen receptor
FMNT	Formononetin
RNA	Ribonucleic acid
GA	Garcinol
GNAT	Gcn5-related N-acetyltransferase
HAT	Histone acetyltransferase
HDAC	Histone deacetylase
MTT	3-(4,5-dimethylthiazol-2-yl)-2,5-diphenyltetrasilium bromide
NAD+	Nicotinamide adenine dinucleotide
NEDD1	Neural precursor cell expressed developmentally downregulated 1
NF-kB	Nuclear factor kappa-light-chain-enhancer of activated B cells
NOD	Non-obese diabetic
PCAF	P300 and p300/CBP-associated factors
PLB	Plumbagin
PTL	Parthenolide
SAC	S-allylcysteine
SAHA	Suberoylanilide hydroxamic acid
SAMC	S-allylmercaptocysteine
SCID	Severe combined immunodeficient
siRNA	Small interfering RNA
SIRT	Sirtuin
